# Extensive Polymorphism in the Molecular Ferroelectric 18-Crown-6 Oxonium Tetrachloro-Gallium(III)

**DOI:** 10.1021/acs.cgd.3c00017

**Published:** 2023-03-23

**Authors:** Sam Y. Thompson, Lauren A. Devenney, Dmitry S. Yufit, John S.O. Evans

**Affiliations:** Department of Chemistry, Science Site, Durham University, South Road, Durham DH1 3LE, United Kingdom

## Abstract

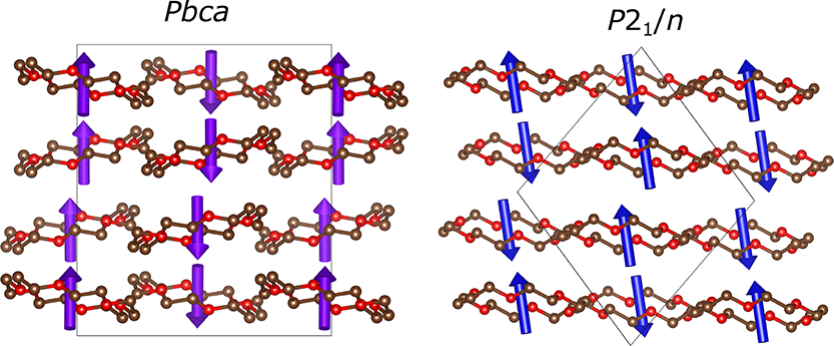

The materials property of ferroelectricity is intimately linked with symmetry-changing phase transitions. Characterizing such transitions is therefore essential for understanding molecular ferroelectrics. In this paper, we explore the temperature and thermal history dependence of polymorphic phase transitions in the multiaxial molecular ferroelectric 18-crown-6 oxonium tetrachloro-gallium(III). We have solved the structures of two previously suggested polymorphs (D and Y) *ab initio* from high-temperature powder diffraction data. We also report the structure of a new polymorph (X) using low-temperature powder diffraction data and identify a fifth (W) that can form on cooling. These polymorphs can be related using two distinct group–subgroup trees. Structure types A–C observed in this and related compounds can be derived from high-temperature polymorph D by group–subgroup relationships. The X and Y polymorphs can be described as child structures of a hypothetical polymorph Z using a molecular rotational distortion mode description. The ferroelectric properties of the various polymorphs can be rationalized based on our structural findings.

## Abstract 2

In this paper, we explore the temperature and thermal history dependence of polymorphic phase transitions in the multiaxial molecular ferroelectric 18-crown-6 oxonium tetrachloro-gallium(III).

## Introduction

Ferroelectric materials with a switchable permanent polarization are used in a range of applications such as capacitors and memories.^1^ The related property of piezoelectricity is exploited in applications such as positioners, transducers, sensors, and energy harvesters.^2^ The most widely used ferroelectrics are inorganic ceramics such as (BaTiO_3_)^3^ and (Pb(*Zr*, *Ti*)O_3_),^4^ which have properties sufficient for many applications. These materials are generally multiaxial, meaning that random domain orientation in thin films does not greatly impact their saturated polarization (*P*_S_): high pseudo-symmetry leads to multiple equivalent polarization directions within each domain. The energy-intensive synthesis of inorganic ceramics, their density, and their toxicity makes alternative materials desirable. Molecular ferroelectrics, which are generally more processable and can be prepared at lower temperatures, are a potential alternative.^5,6^

**Scheme 1:**
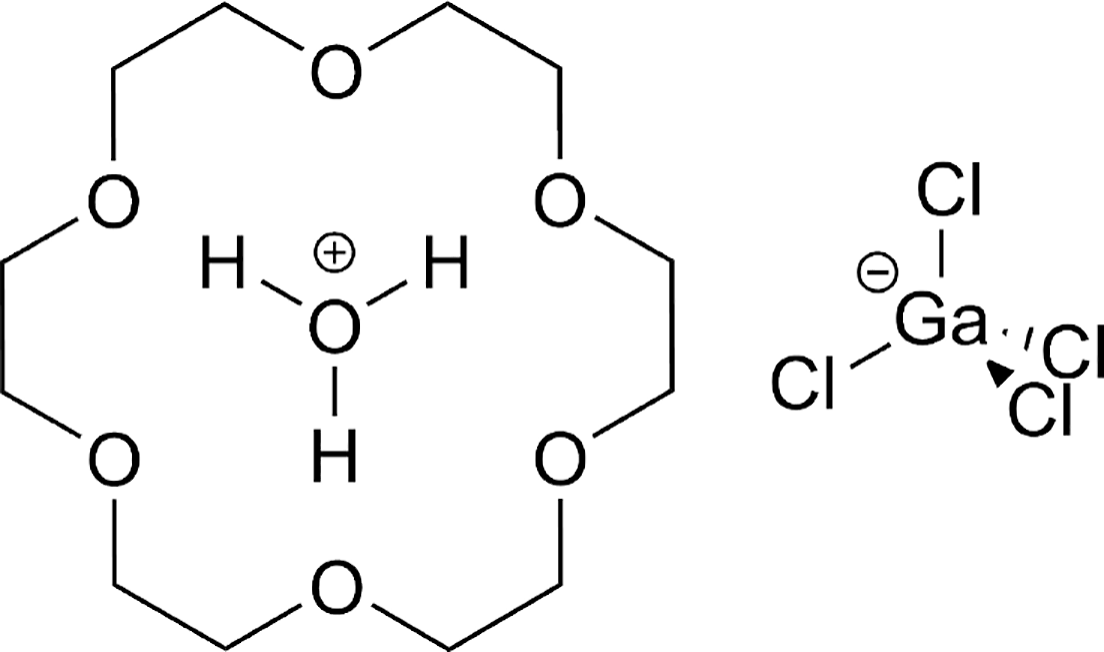
Molecular Structure of **1**

One drawback of many molecular ferroelectrics is the reduced number of polarization directions compared to inorganic ferroelectrics, which could limit their application in thin films. The host–guest inclusion compound 18-crown-6 oxonium tetrachloro-gallium(III) (**1**) (see Scheme 1) was recently reported as an above room-temperature multiaxial ferroelectric with a Curie temperature (*T*_C_) of 337 K and a *P*_S_ of 3.9 μC cm^–2^, and has four polarization directions.^7^ These properties suggest that **1** might be suitable in thin film applications.

The reported room temperature structure of **1** is orthorhombic and polar (space group *Pmc*2_1_) with the polarization arising from a combination of the displacement of GaCl_4_^–^ anions in a 2:1 ratio along the negative and positive *c*-directions and, potentially, the orientation of the oxonium cations within the 18-crown-6 host. We label this polymorph **1**-A for clarity (it was named LTP by Zhang et al.). Other polymorphs reported herein are labeled B, C, and D (Zhang et al.’s HTP) or W, X, Y (Zhang et al.’s ITP), and Z according to their location on different subgroup trees as discussed below. On heating, **1**-A retains ferroelectricity up to 337 K, at which temperature a phase transition occurs to an uncharacterized intermediate phase (**1**-Y). A second phase transition was reported at 352 K, with this third phase (**1**-D) suggested to crystallize in tetragonal space group *P*4_2_/*nnm*; however, a crystal structure was not reported. Upon cooling, a direct transition was observed from **1**-D to **1**-A at 320 K without passing through the intermediate phase, **1**-Y.

Given the interesting ferroelectric properties of **1**, and the apparent difference in phase transition sequences on warming and cooling, we have investigated the polymorphic behavior of **1** using variable temperature powder X-ray diffraction. We have been able to solve the structures of polymorphs **1**-Y and **1**-D. In addition, we have identified a fourth polymorph, **1**-X, and a suggestion of a fifth (**1**-W). We can rationalize the structural changes and their impact on the ferroelectric properties of these polymorphs (and two others reported for related compositions) in terms of group–subgroup relationships. We show how symmetry methods can reveal a close structural relationship between **1**-X and **1**-Y, despite their markedly different powder diffraction patterns.

## Experimental Section

### Synthesis

18-Crown-6 oxonium tetrachloro-gallium(III) was synthesized according to the literature method.^7^ 18-Crown-6 (0.64 g, 2.4 mmol) and gallium trichloride (0.43 g, 2.4 mmol) were dissolved in methanol (5.5 mL) and deionized water (0.5 mL), respectively. The two solutions were combined, and then hydrochloric acid (0.25 mL, 12 M) was added dropwise over 1 min. The reaction mixture was stirred for a further 10 min, and then, the solvent was allowed to evaporate overnight at room temperature, producing colorless block crystals. Polycrystalline samples were prepared by gentle grinding of the crystals.

The iron analogue of **1** was synthesized by dissolving 18-crown-6 (0.64 g, 2.4 mmol) and iron trichloride (0.39 g, 2.4 mmol) in methanol (5.0 mL) and deionized water (1.0 mL), respectively. Both solutions were combined, and then, hydrochloric acid (0.25 mL, 12 M) was added dropwise over 1 min. The reaction mixture was stirred for 10 min and then left uncovered at room temperature to allow the solvent to evaporate. Pale yellow crystals formed after 2 weeks.

### Powder X-ray Diffraction (PXRD)

Variable temperature PXRD data were collected using a Bruker D8 ADVANCE Mo Kα diffractometer, equipped with a LYNXEYE detector and an Oxford Cryosystems Cryostream Plus device. The sample was loaded into a 0.7 mm external diameter borosilicate capillary to a length of 30 mm. The capillary was sealed and attached to a goniometer, which rotated at 10 rotations a minute during the measurements. Data were collected between 80 and 400 K with a heating/cooling rate of 17 K/h. PXRD patterns were recorded every 30 min in the 2θ range of 2–35°, resulting in a pattern being recorded every 10 K. Cryostream temperatures were calibrated using a 1:1 ratio of Al and Si powders, which have significantly different thermal expansion coefficients. Diffraction patterns were collected between 80 and 500 K with the same setup, and the true sample temperatures were determined by comparing experimental cell parameters to known thermal expansion data.^8−10^

### Single-Crystal X-ray Diffraction (SXRD)

SXRD data were collected using a Bruker D8 VENTURE diffractometer (PHOTON III C7 MM CPAD detector, ImS-microsource, focusing mirrors) equipped with an Oxford Cryosystems Cryostream 700+ device using Mo Kα radiation. Crystal structures were solved within the Olex2 software package.^11^ H atoms were placed in calculated positions and refined in riding mode.

### *Ab Initio* Crystal Structure Solution

Structure solution from PXRD data was performed with TOPAS-Academic.^12−14^ Rigid bodies were defined for the 18-crown-6 molecule either using the model in Figure S1 or the disordered ring model discussed later as well as for GaCl_4_^–^ ions. Rigid body positions and rotations were randomized, and a restricted Rietveld refinement was performed. After convergence, the model was re-randomized and refined to convergence for 100,000 least-squares iterations. In each case, the same low R-factor solution was found multiple times. The best structural model was then used in a full Rietveld refinement where peak shapes, atomic displacement factors, rigid body coordinates, cell parameters, and zero-point correction factor were refined.

## Results and Discussion

### Observations of Structural Phase Transitions in **1**

**Figure 1:**
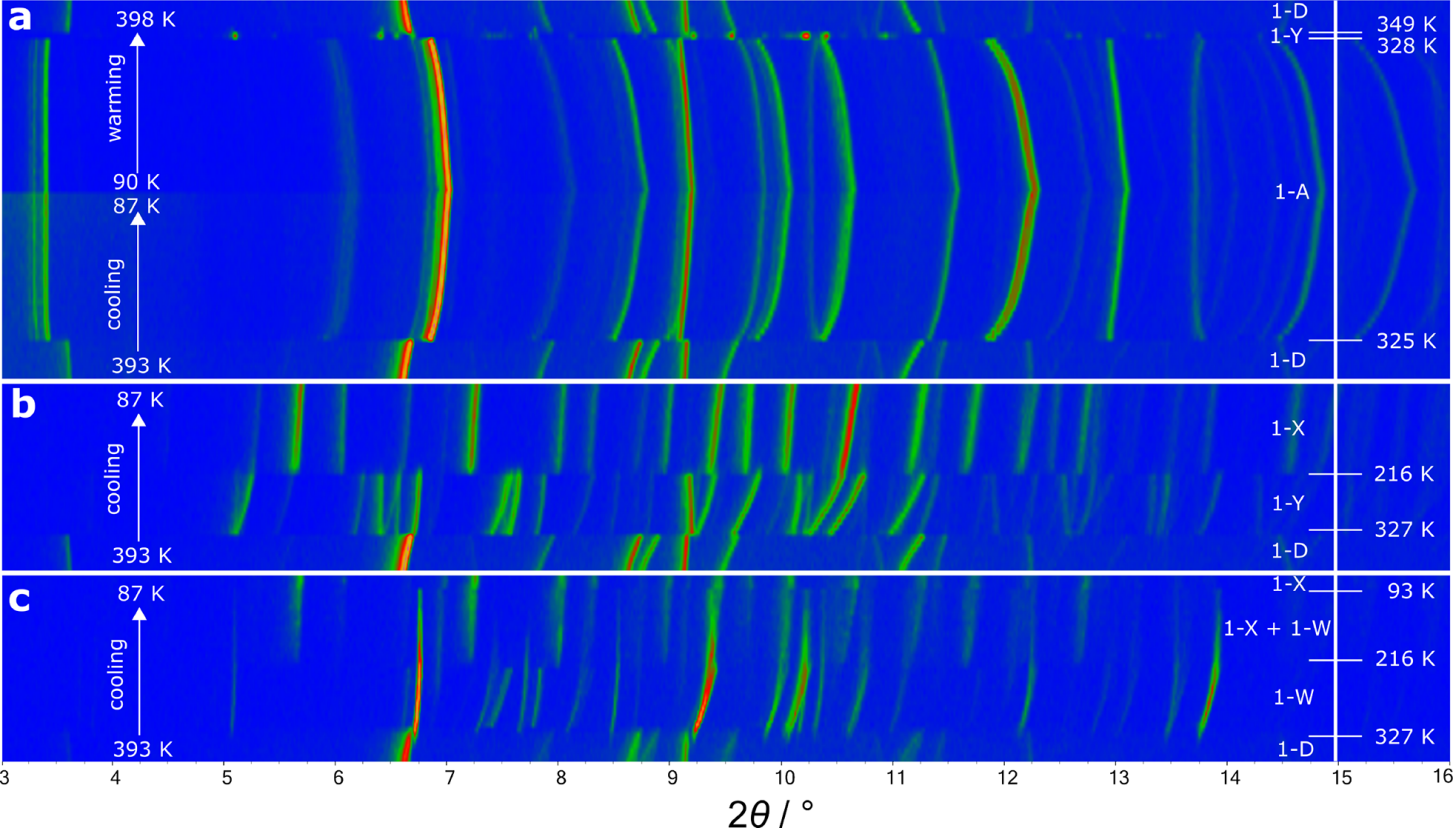
Surface plots of variable temperature PXRD data on (a) cooling and warming between 398 and 87 K, (b) cooling from 393 to 87 K, and (c) cooling from 393 to 87 K. Peak intensities are represented with an artificial color map: low intensity is blue, and high intensity is orange. The rate of change of temperature for all three experiments was 17 K/h.

**Figure 2:**
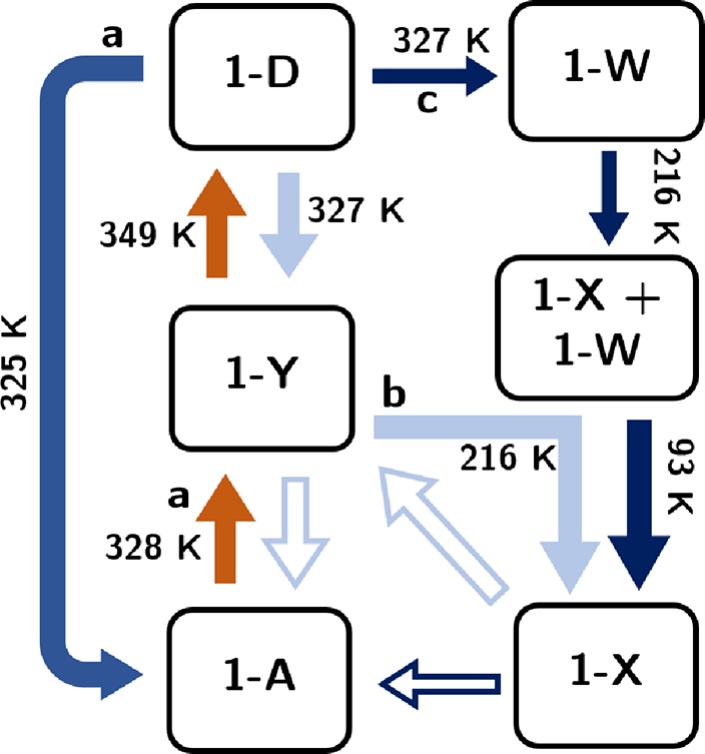
Graphical summary of observed phase transitions for **1**. Data from Figure 1a: orange and medium blue arrows, Figure 1b: light blue, and Figure 1c: dark blue. Hollow arrows are transitions that occurred when samples were held at room temperature for ∼12 h.

To confirm the findings of Zhang et al. on the phase transition sequence of **1**, variable temperature (VT) PXRD data were collected between 80 and 400 K upon warming and cooling at 17 K/h (Figure 1a). The abrupt changes observed in the Bragg peaks were similar to those previously reported: two phase transitions were observed on heating (**1**-A → **1**-Y → **1**-D) and one on cooling (**1**-D → **1**-A). This behavior is summarized in Figure 2. Despite significant shifts in 2θ, there is sufficient similarity between the powder patterns of **1**-A and **1**-D to suggest a close structural relationship between them. The smaller number of Bragg peaks observed in the diffraction pattern of **1**-D compared to **1**-A suggests a higher symmetry phase. The intermediate phase (**1**-Y) has a markedly different diffraction pattern to the other two with the larger number of peaks suggesting a significantly different lower symmetry structure.

When the VT-PXRD experiment was repeated on a fresh sample under essentially identical conditions, different phase transition sequences were observed. In particular, some experiments suggested the formation of new polymorphs. Figure 1b,c shows results from two cooling experiments (again at 17 K/h). In both, exclusively **1**-D is observed at 390 K and a new polymorph we label as **1**-X is observed at 80 K. During the data collection of Figure 1b, a first transition occurred at ∼325 K to **1**-Y (in Figure 1a and in the data reported by Zhang et al., **1**-Y was only seen on heating) and then a second transition to **1**-X at ∼215 K. During the data collection of Figure 1c, different phase evolution was observed between **1**-D and **1**-X. In this experiment, **1**-Y was not observed. Instead, the change in diffraction data suggests a transition to a different polymorph or mixture of polymorphs that we have not been able to identify. We label this composition **1**-W. It is clear from these experiments that the phase evolution of **1** has a high sensitivity to local conditions and thermal history and that more polymorphs are possible than previously reported. Our observations are summarized in Figure 2.

### Crystal Structure of **1**-A

At 93 K, **1** was previously reported in space group *Pmc*2_1_ with cell parameters *a* = 35.430(3) Å, *b* = 8.7631(6) Å, and *c* = 10.0221(7) Å using single-crystal X-ray diffraction (SXRD).^7^ This was confirmed by our own SXRD experiment that gave cell parameters of *a* = 35.517(2) Å, *b* = 8.8019(6) Å, and *c* = 10.1593(7) Å at 120 K and an essentially identical structural model (see CCDC-2234329). The structure contains ordered 18-crown-6 rings and ordered GaCl_4_^–^ tetrahedra (see Figure 5 later).

### Crystal Structure of **1**-D

The polymorph of **1** found above 352 K was previously suggested to crystallize in tetragonal space group *P*4_2_/*nnm* with cell parameters *a* = *b* = 10.3428 Å and *c* = 11.3392 Å at 353 K. These parameters were derived from low-quality SXRD data and refined against PXRD data using the structure-independent Pawley method.^15^ Since crystals of **1** invariably shatter through the **1**-A → **1**-Y → **1**-D transitions, we have used powder methods to solve its structure. Our analysis confirms the tetragonal cell, but Pawley refinements against 398 K PXRD data gave an *R*_wp_ of 20.9% using *P*4_2_/*nnm*. Several significant Bragg peaks were not predicted, as seen in Figure S2. The systematic absences [e.g., (001), (201)/(021), and (210) are missing] are consistent with the extinction conditions of *P*4*bm*, *P*4̅*bm*, and *P*4/*mbm*. Individual Pawley refinements using these space groups resulted in comparable *R*_wp_ values of around 5%, a much better fit than with *P*4_2_/*nnm*. *P*4/*mbm* was chosen for further analysis as it is the only space group that is related to that of **1**-A by a straightforward group–subgroup relationship.

Due to the high symmetry of *P*4/*mbm* and the high temperature at which **1**-D is observed, a significant amount of disorder is expected in the molecular components. This is supported by the large entropy increase at the transition of 45.6 J K^–1^ mol^–1^ as calculated from differential scanning calorimetry data by Zhang et al. GaCl_4_^–^ was initially approximated as spherically disordered and 18-crown-6 as flat rings. The GaCl_4_^–^ disorder was modeled with a spherical distribution of 128 chlorine atomlets at a refinable radius around a central gallium atom situated on Wyckoff site 2*a*. The 4/*m* site symmetry is satisfied by this essentially spherical anion. The disordering of tetrahedral anions to higher symmetries is common in this type of compound.^16−19^

**Figure 3:**
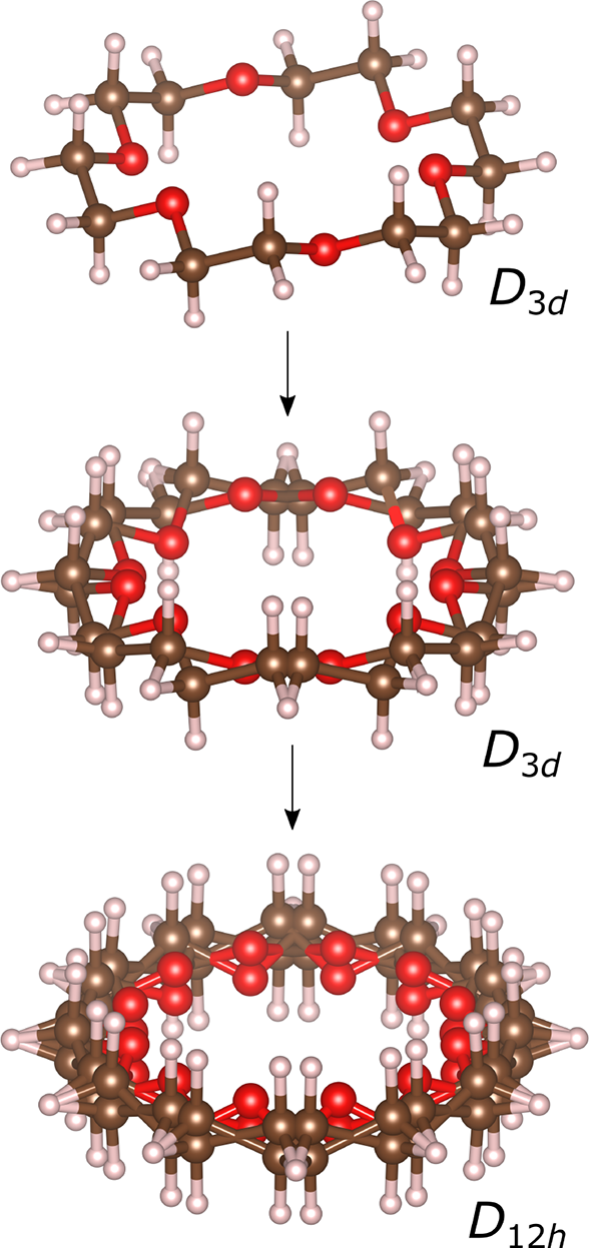
Molecular disorder model for 18-crown-6. Site symmetry increases via a “merry-go-round” rotational mechanism.

To satisfy the 4-fold axis of the space group, adjacent 18-crown-6 rings must have their mean planes perpendicular to one another, which is possible by placing their center on Wyckoff position 2*d*. The 2*d* site symmetry is *mmm*, whereas the 18-crown-6 molecule has 3̅*m* (*D*_3*d*_) point symmetry. This point symmetry incompatibility implies significant disorder. Fast merry-go-round rotational disorder of 18-crown-6 in the solid state has been studied previously,^20^ including in the context of ferroelectrics.^19^ This mechanism increases the point group symmetry to 12/*mmm* (*D*_12*h*_), which lowers to *mmm* in its crystal environment. Figure 3 sketches this disorder. The disordered 18-crown-6 molecules were therefore modeled by eight rings of atomlets: one carbon, one oxygen, and two hydrogen plus symmetry-generated rings. The oxonium ion in the center of the ring was modeled as being rotationally disordered in the plane of the ring.

**Figure 4:**
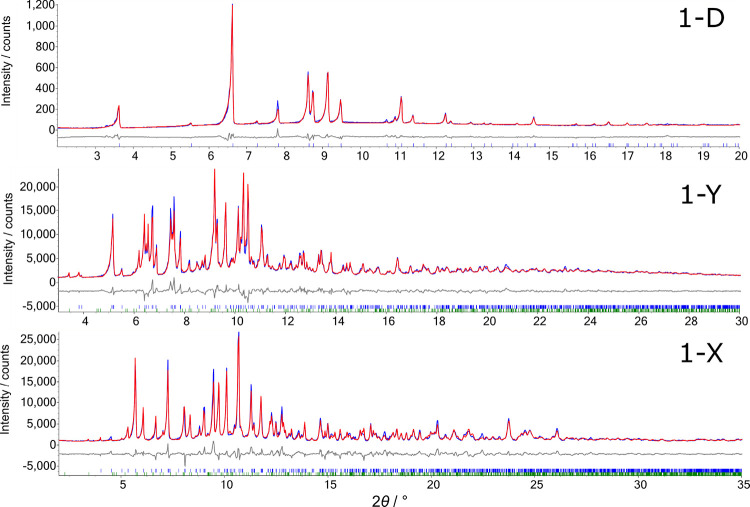
Powder X-ray diffraction data (blue) of **1** showing the calculated pattern from the Rietveld refinement (red) and the difference curve (gray) for **1**-D at 398 K, **1**-Y at 281 K, and **1**-X at 87 K.

The structure was solved by repeated cycles of randomization and Rietveld refinement in which the ring radius and thickness as well as the Ga–Cl sphere radius were varied. In a second step, the occupation of the Cl atomlets was also allowed to refine. Those that refined to a non-zero occupancy could be described using a single GaCl_4_^–^ tetrahedron that generated seven other orientations through the symmetry elements of the space group. A difference Fourier map calculated based on the resultant structure suggested a small region of excess electron density along the *c* axis of the unit cell. This was modeled using an oxygen atom at (0, 0, 0.5) and was assumed to be a site partially occupied by oxonium ions at high temperature. The final *R*_wp_ of the Rietveld refinement was 6.15% with cell parameters *a* = *b* = 10.3782(3) Å and *c* = 11.1734(5) Å. The Ga–Cl bond distance refined to 2.28(2) Å at 398 K, slightly higher than the mean value from a sample of 114 literature structures: 2.163 Å. This higher value is reasonable considering the high temperature at which this phase exists. The Rietveld plot is shown in Figure 4a. The overall agreement between observed and calculated patterns is good for a highly dynamically disordered system, giving confidence in the model. Minor discrepancies such as the mismatch at 7.8° 2θ could be caused by diffuse scatter, and we note that this peak shows a similar discrepancy in the structure-independent Pawley fit of Figure S2.

### Crystal Structure of **1**-Y

In the VT-PXRD experiment of Figure 1a, the intermediate phase **1**-Y was observed on heating over a range of less than 10 K. This meant that no single pattern exclusively contained **1**-Y, making structure solution difficult. The phase could, however, be isolated in a subsequent experiment in which a sample was cooled from 390 to 80 K at 17 K/h, forming **1**-X. The sample was warmed to 150 K at 60 K/h, and a transition to **1**-Y occurred between 102 and 144 K. The sample was then heated to 281 K, and data were collected for 24 h, giving a high-quality data set from which the structure could be solved.

Through observation alone, **1**-Y appears structurally unrelated to **1**-D and **1**-A: it does not seem to share obvious peak intensity patterns with either of these phases, and its powder pattern is markedly more complex than **1**-A, indicating a lower symmetry. The transition from higher symmetry in **1**-A to lower symmetry in **1**-Y on increasing temperature is rare but not unprecedented.^21^ The pattern was indexed,^22^ giving a monoclinic cell with space group *P*2_1_/*n*. The cell parameters obtained were similar to a phase seen between 347 and 364 K in the iron analogue of **1**(23) (structural details for this phase were not reported). A Pawley refinement gave an *R*_wp_ of 4.20% and is shown in Figure S3. As discussed below, a small number of weak peaks not predicted by this cell could be explained by the presence of a small amount of **1**-A. The refined unit cell dimensions were *a* = 8.6747(4) Å, *b* = 20.7290(12) Å, *c* = 12.7033(8) Å, and β = 103.248(4)°.

**Figure 5:**
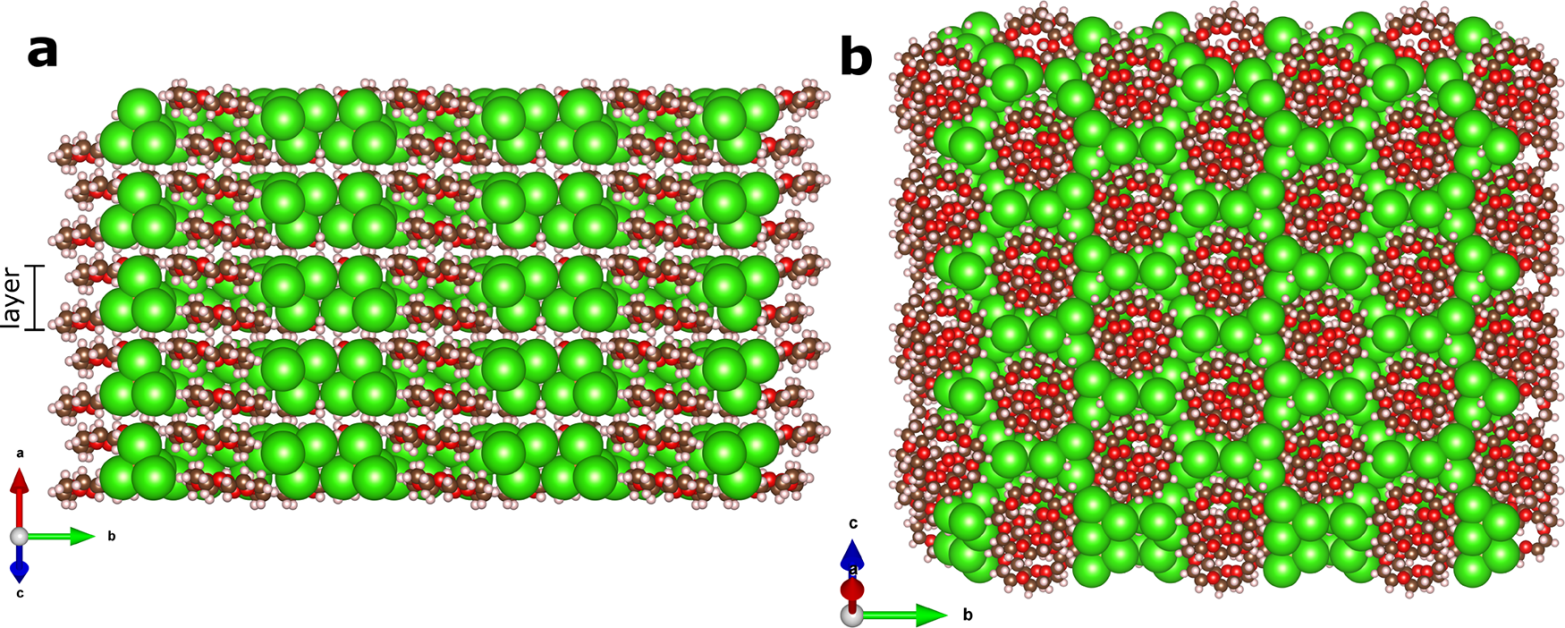
Space-filling projections of the crystal structure of **1**-Y showing (a) side-on and (b) top-down views of the GaCl_4_^–^ layers. Colored spheres show chlorine (green), carbon (brown), oxygen (red), and hydrogen (pink) atoms.

Structure solution was performed using TOPAS-Academic. Rotational and translational degrees of freedom for 18-crown-6 and GaCl_4_^–^ rigid bodies were randomized and refined over multiple cycles. An extra degree of freedom describing the radius of the 18-crown-6 ring was also refined. The best model was used in a Rietveld refinement. Unfitted peaks at 3.41, 6.87, 9.84, and 11.93° suggested the presence of a small amount of **1**-A. An inclusion of 8.9 wt % of **1**-A to the model gave the Rietveld plot of Figure 4b and an *R*_wp_ of 5.82%. The final structure is, as anticipated, not structurally related in any obvious way to **1**-A or **1**-D. The structure can instead be described as containing mixed layers of GaCl_4_^–^ tetrahedra and 18-crown-6 molecules, as shown in Figure 5. GaCl_4_^–^ tetrahedra are arranged to give the approximately hexagonal rings of Cl shown in Figure 5b. Each tetrahedron has a 3-fold axis approximately perpendicular to the layers with three “up” and three “down” in each ring. As such, the Cl atoms lie in two distinct layers in the view of Figure 5a. The 18-crown-6 molecules are located inside the Cl rings, approximately co-planar with the Cl outer layers. In Figure 7, we depict just the Ga network for simplicity. The Ga layers themselves are slightly buckled, forming a 2D net of fused chair-cyclohexane-like rings reminiscent of the layers in black phosphorus. The two 18-crown-6 molecules associated with each ring lie above and below the mean Ga plane. The layer stacking sequence is discussed later. The centrosymmetric nature of **1**-Y is consistent with the loss of second harmonic generation (SHG) activity at 337 K.^7^

This same structure was subsequently found (using SXRD at 345 K) for the iron analogue (see CCDC-2234330). This showed cell parameters of *a* = 8.7508(4) Å, *b* = 20.7464(10) Å, *c* = 12.7982(6) Å, and β = 103.904(2)° at 345 K. Details can be found in the Supporting Information.

### Crystal Structure of **1**-X

The structure of **1**-X was solved from a summation of 34 30 min scans at 87 K taken from the VT-PXRD experiment of Figure 1b. The pattern was indexed, and Pawley refinements were carried out using several possible space groups, with *Pbca* giving a good fit to the data with *R*_wp_ = 3.44% and explaining all the strong reflections. Weak additional reflections were again accounted for using the cell of **1**-A in a two-phase Pawley refinement (Figure S3). Unit cell parameters refined to *a* = 20.1512(15) Å, *b* = 13.3276(11) Å, and *c* = 15.2087(11) Å. *Ab initio* structure solution was performed in a similar manner to **1**-Y. The final Rietveld refinement (Figure 4c) gave an *R*_wp_ of 11.22%. A small number of weak unfitted peaks can again be attributed to around 4.7 wt % of **1**-A. A single-unit cell of **1**-X is shown alongside that of **1**-Y in Figure S5. Despite the significant difference in their PXRD patterns, there are strong similarities between these structures. **1**-X can again be described as containing layers of GaCl_4_^–^ tetrahedra that create pseudo-hexagonal rings in which the 18-crown-6 molecules are located, though the layer thickness and layer stacking patterns differ to **1**-Y. The Ga net is again similar to the layers found in black phosphorus. These black phosphorus-like layers are not present in **1**-A or **1**-D, suggesting that **1**-X and **1**-Y are related to one another but distinct from **1**-A and **1**-D. The structural relationship between **1**-X and **1**-Y is discussed in more detail later.

### Structure Relationship between **1**-A and **1**-D and Implications for Ferroelectricity

The similarity in the diffraction patterns of **1**-A and **1**-D implies a structural relationship between the two. A convenient way to describe the relationship is using the language of isotropy subgroups.^24^*Pmc*2_1_ is a subgroup of *P*4/*mbm* via an intermediate *Pbam* subgroup. *Pmc*2_1_ itself can then distort to a tripled cell. The proposed subgroup tree is thus *P*4/*mbm* → *Pbam* → *Pmc*2_1_ → *Pmc*2_1_ (**a′** = 3**a**). This is consistent with the VT-PXRD data on cooling, which shows an abrupt change in the diffraction patterns from *P*4/*mbm* to *Pmc*2_1_ (**a′** = 3**a**). This first-order character of the phase transition is required as multiple irreducible representations are active during the transition.

The two intermediate structures, *Pbam* and *Pmc*2_1_ without unit cell tripling, have not been observed for **1**; however, both structures are known in the literature for closely related compounds. Replacing GaCl_4_^–^ with PF_6_^–^, TaF_6_^–^, NbF_6_^–^, or AsF_6_^–^ gives compounds adopting the *Pbam* structure.^25−27^ In these, the high symmetry of the O_h_ anions allows *Pbam* symmetry, whereas T_d_ anions break the *a* glide plane and the symmetry lowers to *Pmc*2_1_. Replacing GaCl_4_^–^ with FeCl_4_^–^ gives a *Pmc*2_1_ structure without the cell tripling at room temperature.^23,28^ The energetic balance between these structures must be subtle. Analysis of the CSD shows that GaCl_4_^–^ and FeCl_4_^–^ have similar mean sizes (mean metal to chlorine bond lengths, respectively, are 2.163 Å s.d. 0.019 Å from 114 structures and 2.192 Å s.d. 0.067 Å from 360 structures at 293 ± 10 K) and a similar degree of distortion with standard deviations of tetrahedral angles of 3.0 and 2.9°, respectively. The slightly larger range of Fe–Cl distances implying higher tetrahedral flexibility might help stabilize the untripled structure.

**Figure 6:**
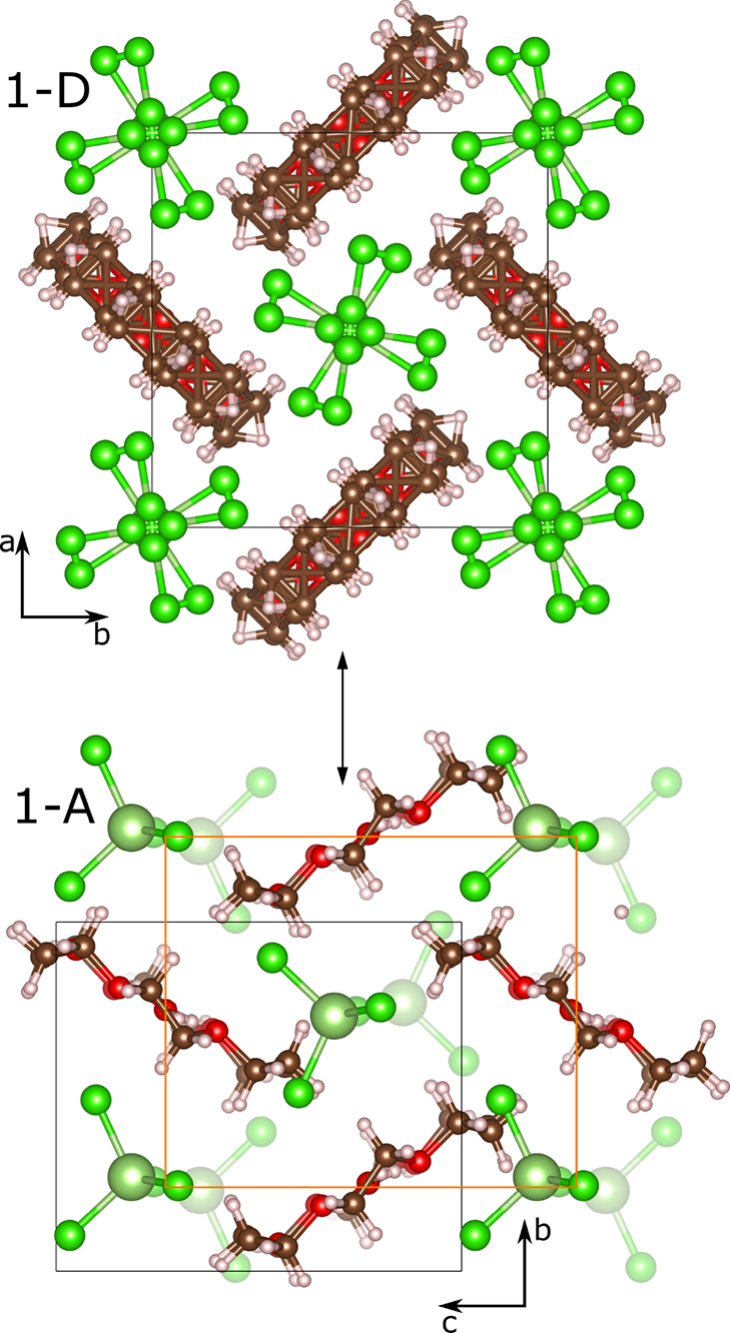
Structural relationship between **1**-A and **1**-D. The orange square in **1**-A shows the **1**-D unit cell with its origin shift.

There are thus multiple processes that occur during the phase transition between **1**-D and **1**-A. Figure 6 summarizes these graphically. The 18-crown-6 rings order from an average planar structure due to the loss of dynamic disorder, and the inter-ring angle changes from 90 to 59.2°. The GaCl_4_^–^ anions order from eight orientations at high temperature to a single orientation while displacing along the *c* axis (of **1**-A) to cause the tripling of the unit cell. The oxonium ion also orders from multiple positions at high temperature to discrete orientations at low temperature (from the SXRD structure of Zhang et al.).^7^

In ferroelectric **1**-A, the source of the polarization is largely due to the separation of charges along the *c* axis between the GaCl_4_^–^ anions and the H_3_O^+^ cations at the center of the 18-crown-6 rings. This displacement can be seen in Figure 6 where there is a ratio of 2:1 between the faded and unfaded GaCl_4_^–^ ions drawn. The ferroelectric switching mechanism involves the displacement of the GaCl_4_^–^ ions such that this ratio becomes 1:2. Assuming that the H_3_O^+^ oxygen atoms and Ga atoms are the center of postive and negative charge, respectively, the unit cell polarization is estimated as 0.85 μC cm^–2^. The loss of polarization at *T*_C_ is due to the displacement of both faded and unfaded GaCl_4_^–^ ions onto the corners of the orange unit cell, thus creating an inversion center as the crystal transitons to a non-polar space group. The ordered oxonium ions in **1**-A could also contribute to the polarization of the unit cell by rotating during ferroelectric switching. Their disordering creates another inversion center.

The multiaxial properties of **1** arise from the large symmetry increase between **1**-A and **1**-D. The ratio of symmetry elements between *P*4/*mbm* and *Pmc*2_1_ is 4, which indicates four polarization directions in **1**-A. The pseudo-tetragonal structure of **1**-A (*b* ≈ *c*) means that ferroelectric switching could occur along more directions than just the polar axis *c* of *Pmc*2_1_ (two polarization directions). Effectively, an electric field perpendicular to the polar axis rearranges the space group to *Pm*2_1_*b* and *b* becomes the polar axis (two further polarization directions).

### Structural Relationship between **1**-Y and **1**-X

**Figure 7:**
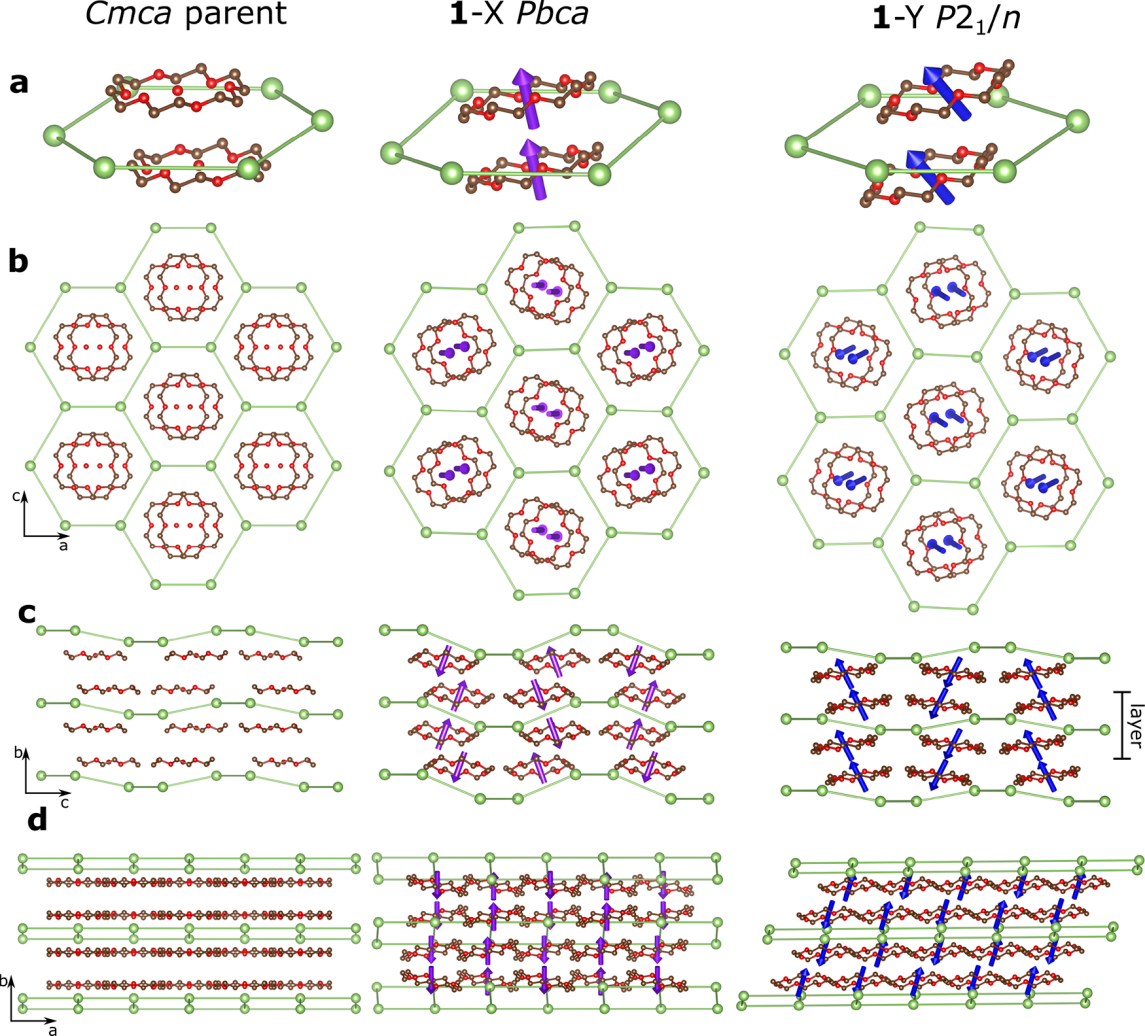
Structural relationship between **1**-X and **1**-Y and a hypothetical *Cmca* parent structure. (a) 18-Crown-6 pair and its pseudo-hexagonal unit. (b) Viewing a single layer along the *b* axis shows the pseudo-hexagonal structure. (c) Viewing along the *a* axis shows the increased buckling of the Ga layers in **1**-X and the identical rotations of 18-crown-6 pairs. (d) Viewing along the *c* axis shows the increased buckling in **1**-X and the sliding layers in **1**-Y due to the strain mode of the distortion. Hydrogens and chlorines are omitted for clarity. Non-bonded Ga–Ga contacts are drawn to demonstrate the hexagonal-like arrangement of their packing. The rotation vectors are drawn so that 1 Å = 25.9°.

The abrupt changes in the diffraction pattern in Figure 1b between **1**-Y and **1**-X suggest a first-order phase transition. Due to the similar structural features, notably the pseudo-hexagonal black phosphorus-like layers discussed previously, a structural relationship between these two phases seems likely. In fact, it is possible to relate both structures to an unobserved hypothetical structure in space group *Cmca* with cell parameters similar to those of **1**-X. We note that this is the same space group as black phosphorus, and the Ga atomic arrangements in **1**-X and the P of black phosphorus are equivalent. Both observed structures are maximal subgroups of this parent. This hypothetical structure is made up of GaCl_4_^–^ in a buckled black-P-like grid, as shown in Figure 7b. The 18-crown-6 molecules are positioned on either side of the center of each Ga ring with a center-to-center distance of 4.8 Å. The 18-crown-6 molecules lie in the *ac* plane. This planar structure contrasts with the herringbone-like packing of 18-crown-6 molecules in **1**-A and **1**-D (rings at 59.2 and 90°, respectively). Figure 7 shows the relationship between this hypothetical parent and the two observed structures. For consistency in structural descriptions across changing basis vectors, all directions are described relative to the *Cmca* parent.

The structure of **1**-X can be described by the superposition of Y_2_^+^ distortion modes on the *Cmca* parent structure.^29^ This distortion involves a change in the buckling of the hexagonal layers as half the GaCl_4_^–^ ions displace in the positive *b*-direction, and half displace in the negative *b*-direction. In contrast, the structure of **1**-Y can be described by the superposition of Γ_2_^+^ distortion modes on the *Cmca* parent structure. The buckling of the Ga grid is effectively unchanged between the hypothetical *Cmca* parent and **1**-Y; however, the associated Γ_2_^+^ strain mode slides the layers across one another, resulting in displaced layers as the symmetry lowers from orthorhombic to monoclinic.

One way to capture the differences between **1**-X and **1**-Y is through the use of rotational symmetry modes.^21,30,31^ This method places an axial vector at a point in a rigid body description of a molecule (in this case, the centroid of the 18-crown-6 ring). The shift in vector origin between parent and child describes the shift of the molecular centroid, the vector length describes the amplitude of rotation, and the vector direction the single axis around which the molecule is rotated (following the right-hand rule). The ring parent–child relationship is thus fully captured in a single vector, which can be shown graphically.

**Figure 8:**
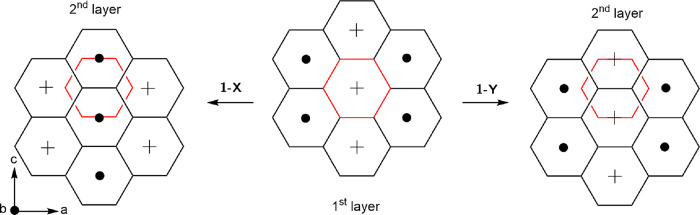
Distribution of rotation vectors within the pseudo-hexagonal layers in crystal structures on subgroup tree 2. Red hexagons show the fixed position of a single hexagon in layer 1. Crosses and dots represent rotation vectors into and out of the page, respectively, for the 18-crown-6 pair relating to each pseudo-hexagon.

The relationships between 18-crown-6 molecules in the *Cmca* parent and **1**-X and **1**-Y child structures are shown by the purple and blue arrows in Figure 7a. Ring rotations are 45.4 and 50.2° in **1-**X and **1**-Y, respectively. The major rotations for both are about the parent *b* axis, parallel to the principal axis of the 18-crown-6 molecule. Each molecule rotates in the same direction as its pair across a Ga_6_ ring, as required by the inversion center at each ring centroid in both subgroups. Note that as these are axial vectors, they “point” in the same direction after inversion. In **1**-X, there is a component of rotation along the *c* axis, which causes the tilting seen in Figure 7d. The component of rotation along the *a* axis is close to zero. In **1**-Y, there is a small component of rotation in both the *a*- and *c*-directions. The clearest distinction between the two structures can be obtained by adopting the language used to describe the ordering of magnetic moments, which are also axial vectors. It is important to note that this is purely a linguistic choice and has no bearing on the magnetic properties of these materials; all are diamagnetic. Focusing initially on a 18c6-Ga-18c6 layer, in both **1**-X and **1**-Y, we see chains of ferromagnetic-like molecules parallel to *c*, with adjacent chains along *a* ordered in an antiferromagnetic sense. The difference in the structures is the way in which one of these layers relates to the next and can be seen most clearly in Figure 7c. In **1**-X, the rotation direction is flipped between adjacent layers along *b*, leading to 18-crown-6 columns with a mixture of ferro- and antiferro-like rotations. In **1**-Y, the corresponding columns show ferro-like rotation with adjacent columns along *a* rotated in an antiferro sense. This is sketched in Figure 8.

Figure 7d also shows that the thickness of the individual layers differs between **1**-X and **1**-Y. In **1**-X, the Cl–Cl outer distance is 8.02 Å compared to 7.60 Å in **1**-Y. The layer stacking sequence in **1**-X is also such that the Cl in the Ga–Cl bond pointing “up” in one layer points directly toward the center of the 18-crown-6 molecule above it. This could be driven by favorable hydrogen bonding interactions between Cl^–^ and ordered oxonium ions at low temperatures.

The first-order nature of the phase transition between **1**-Y and **1**-X is required by the fact that they are on the same level of the subgroup tree (they both have an index of 2 relative to the *Cmca* parent), and so, it is impossible to relate the two structures via a single irrep, i.e., they are not related as a minimal/maximal subgroup pair. The phase transiton from **1**-X to **1**-Y involves a decrease in the buckling of the hexagonal layers of GaCl_4_^–^ that also slide relative to one another due to the active lattice strain mode. The 18-crown-6 pairs are affected in one of two ways. Half of the rings are displaced along the *a*-direction due to the strain mode and also rotate about the *b* axis. The other half do not displace and rotate about the *c* axis. An animation of this transition can be found in the Supporting Information.

### Rationalization of VT-PXRD Data

**Figure 9:**
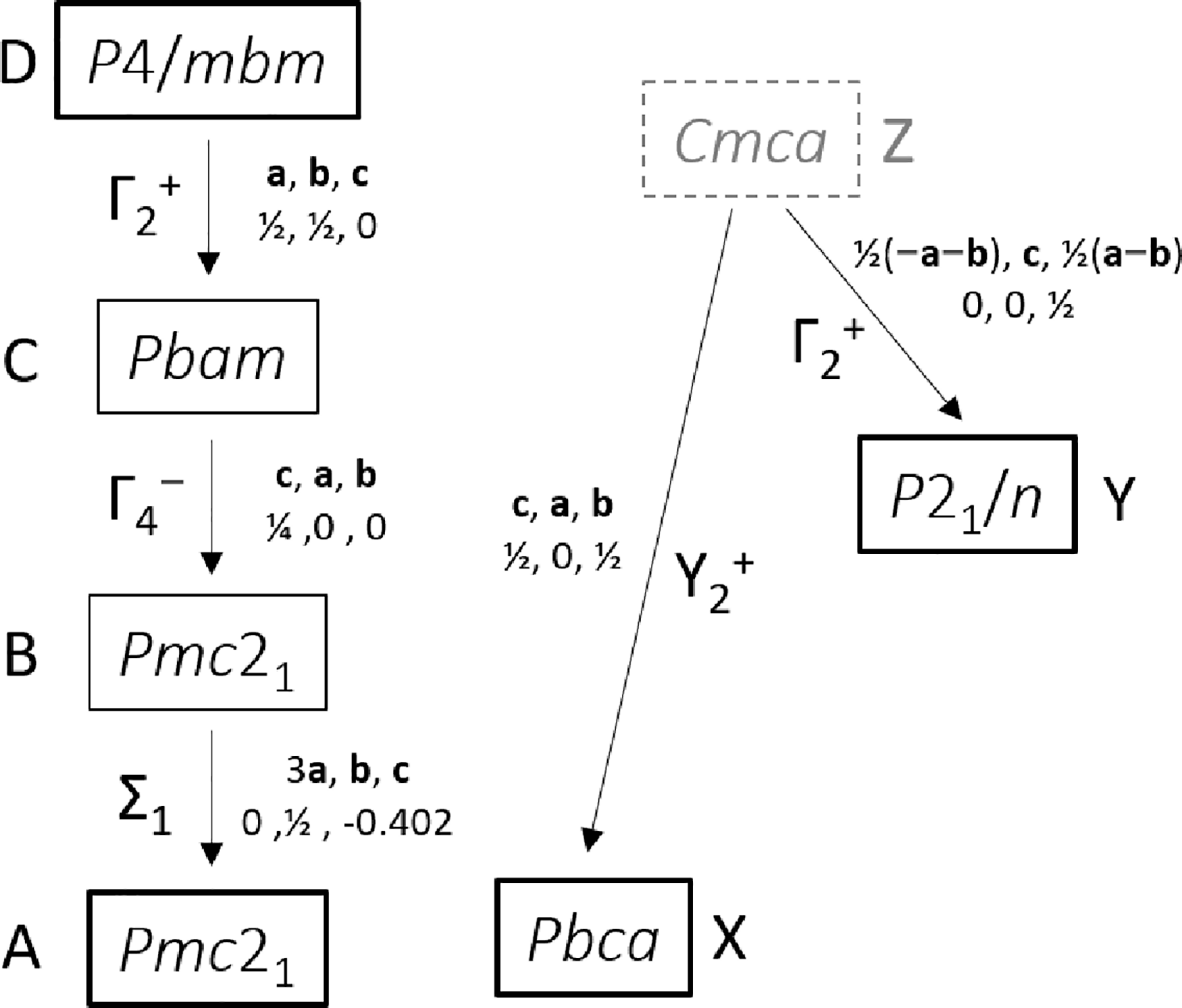
Subgroup trees for thermodynamically stable subgroup tree 1 (left) and (mainly) kinetically stable subgroup tree 2 (right). Bold boxes indicate phases observed for **1**. The irreducible representation associated with each symmetry lowering is shown.

Overall, the observed polymorphs of **1** can be represented on two distinct subgroup trees (Figure 9). Four distinct polymorphs are observed (A, D, X, and Y) for **1** with a further two (B and C) being known in related compositions.

We can use the relationship between them to rationalize the complex sequence of phase transitions observed experimentally in Figure 1. In the VT experiment of Figure 1a, the brief appearance of **1-**Y on warming suggests that it is the thermodynamically stable phase at around 340 K. At 352 K, the temperature is high enough for the GaCl_4_^–^ and 18-crown-6 components to disorder and so a phase transition occurs back to subgroup tree 1 (**1**-D). The high molecular disorder in the larger unit cell of **1**-D (Δ*V* ≈ +2% per formula unit between **1**-Y and **1**-D and Δ*V* ≈ +1% per formula unit between **1**-A and **1**-Y) presumably leads to entropic stabilization. Due to the thermal hysteresis of first-order phase transitions, on cooling **1**-D converts directly to **1**-A at 320 K. This is below the temperature that **1**-Y formed on heating, and **1**-Y is thus not observed. This is the general behavior reported by Zhang et al.

In the VT-PXRD experiment of Figure 1b, cooling a fresh sample from 390 K again resulted in the disappearance of **1**-D at ∼315 K. However, rather than forming **1**-A, as expected, **1**-Y was formed. Since we know that the Figure 1a experiment was kinetically controlled, this different behavior is reasonable. In addition, the sample used for Figure 1b contained trace quantities of **1**-Y prior to heating. It is possible that some of this phase remained outside the hot zone of the capillary that is probed in the VT-PXRD experiments and could have acted as a seed for the growth of **1**-Y on cooling. As the temperature was further decreased, the sample becomes trapped on subgroup tree 2 and transitions to **1**-X on further cooling to ∼215 K, despite these phases being thermodynamically less stable at low temperature than those on subgroup tree 1. Heating to 102–141 K returns the sample to **1**-Y, and leaving overnight at 298 K results in a transition to **1**-A. This suggests that **1**-A is the thermodynamically stable form at room temperature.

The cooling run shown in Figure 1c, which used the same sample as that of Figure 1b, was broadly similar in that **1**-D ultimately transformed to essentially pure **1**-X by 87 K. The behavior between ∼332 and 96 K was, however, different. The pattern contains a mixture of relatively broad and relatively sharp peaks. Several are at 2θ values close to those in either the **1**-D or **1**-X forms, while others appear distinct. This region is bounded by polymorphs **1**-D and **1**-X, and there is no evidence of any change in composition in this temperature range (e.g., TGA data^7^ shows no loss of water). This therefore suggests the presence of a fifth polymorph of **1** (**1**-W) or a mixture of polymorphs in this region. It is possible that this polymorph contains layers similar to those in **1**-X and **1**-Y but with a different interlayer stacking. The data available are not of sufficient quality to make a definitive conclusion.

## Conclusions

Using variable temperature powder X-ray diffraction data, we have solved the crystal structures of three new phases of 18-crown-6 oxonium tetrachloro-gallium(III) using *ab initio* powder diffraction methods. The four polymorphs, as well as some analogous structures from the literature, are related in two distinct group–subgroup trees. Subgroup tree 1 seems to be thermodynamically more stable across most of the temperature ranges studied (87–393 K), with subgroup tree 2 being stable in a small temperature window around 328–349 K. Phases on subgroup tree 2 can be kinetically stabilized to lower temperatures but transform back to subgroup tree 1 at room temperature. All structures observed to date on subgroup tree 2 are centrosymmetric, and ferroelectricity will therefore only be possible on the more stable subgroup tree.

The transition from **1**-A to **1**-D involves significant rotations and displacements of 18-crown-6 rings as well as disordering of GaCl_4_^–^ anions. Structures A–D on subgroup tree 1 differ significantly from X–Z on subgroup tree 2 in that they derive from parents with neighboring 18-crown-6 molecules packed perpendicular and parallel to each other, respectively. The relationship between **1**-X and **1**-Y can be described using rotational symmetry modes to compare both structures to a hypothetical *Cmca* parent structure. This method is effective at relating two otherwise apparently unrelated structures with markedly different PXRD patterns.

## Supporting Information Available

Animation of the structural transition between **1**-X and **1**-Y suitable for viewing in ISOVIZ (TXT)
Molecular structure of the 18-crown-6 rigid body, Pawley refinements for all phases, space-filling projections of **1**-X and **1**-Y, single-unit cell structures of **1**-X and **1**-Y, table of known similar structures, Rietveld refinement tables, and SXRD refinement and fractional coordinates (PDF)
