# Supplementary material for: Extensive Polymorphism in the Molecular Ferroelectric 18-Crown-6 Oxonium Tetrachloro-Gallium(III)
Source: Cryst Growth Des. 2023 Mar 23;23(4):2860–9. doi: 10.1021/acs.cgd.3c00017 (PMC10080656; doi:10.1021/acs.cgd.3c00017)
Supplement: Supplementary file 2 — cg3c00017_si_002.pdf [file cg3c00017_si_002.pdf]

Extensive polymorphism in the molecular ferroelectric 18-crown-6 oxonium tetrachloro-gallium(III)

Sam Y. Thompson, Lauren A. Devenney, Dmitry S. Yufit and John S.O. Evans*

Department of Chemistry, Durham University, Science Site, South Road, Durham DH1 3LE, United Kingdom

Supplementary information

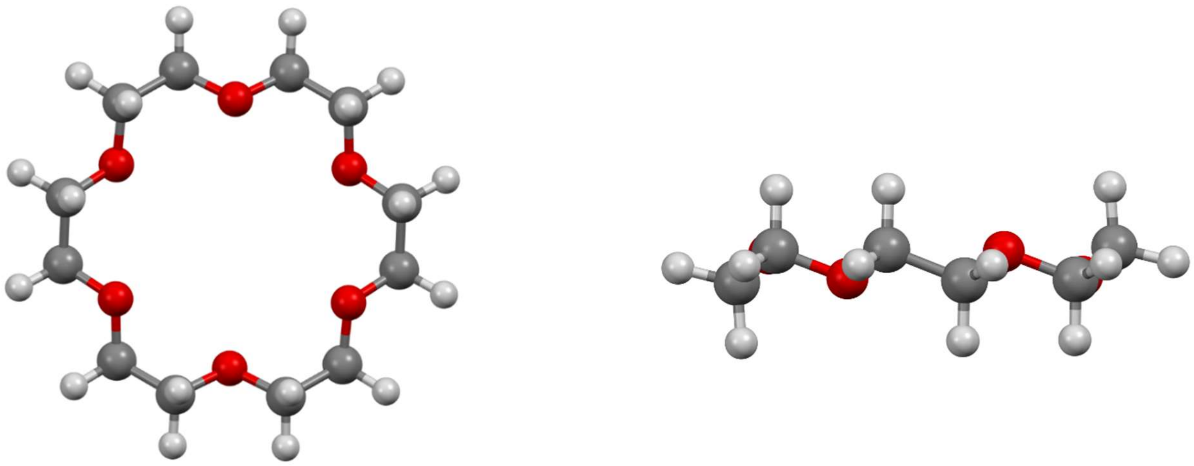

Figure S1 - Rigid body description of 18-crown-6 used in structure solutions. The structure is a result of a global minimum conformational search in Scigress on 18-crown-6 resulting in the D_{3d} conformation generally found in the solid state when 18-crown-6 is host to a guest molecule.

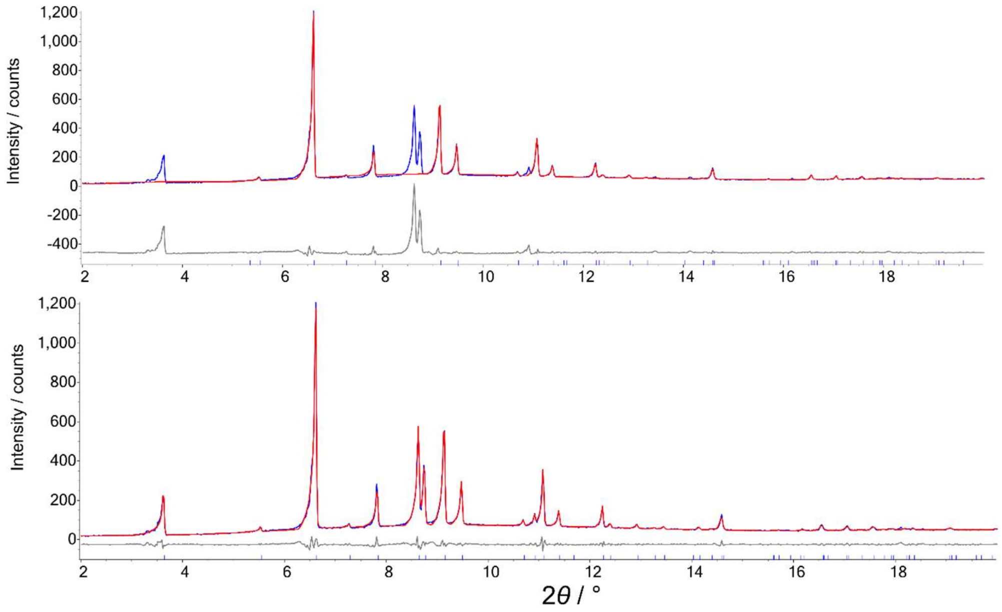

Figure S2 – Pawley refinements on PXRD data recorded at 398 K using previously reported space group $P4_2/nnm$ (top) and space group $P4/mbm$ (bottom).

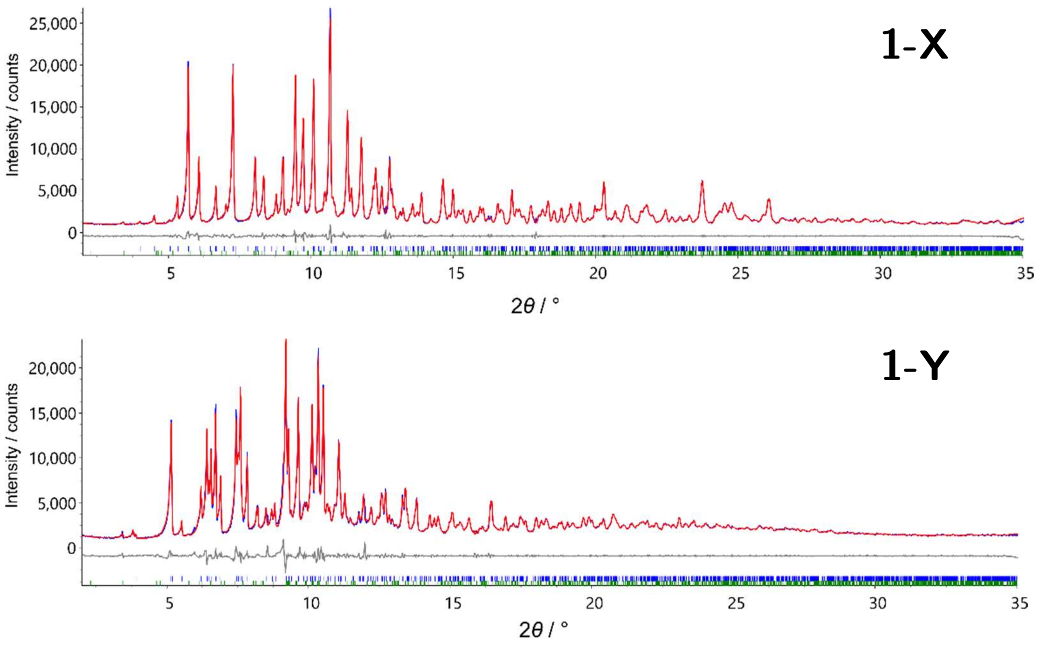

Figure S3 – Two-phase Pawley refinements on PXRD data recorded at 87 K using **1**-X and **1**-A cells (top) and at 281 K using **1**-Y and **1**-A cells (bottom).

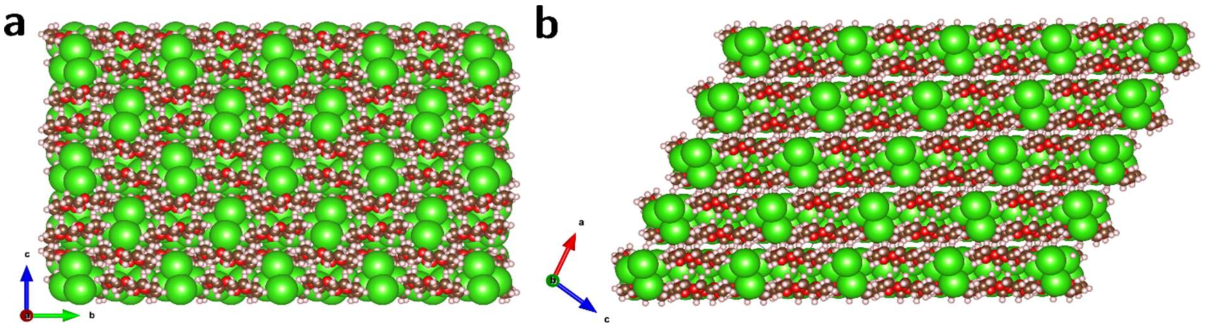

Figure S4 – Space-filling projections of the crystal structure of **1**-X (a) and **1**-Y (b) showing the difference in layer sliding caused by the lattice strain mode in **1**-Y. Coloured spheres show chlorine (green), carbon (brown), oxygen (red) and hydrogen (pink) atoms.

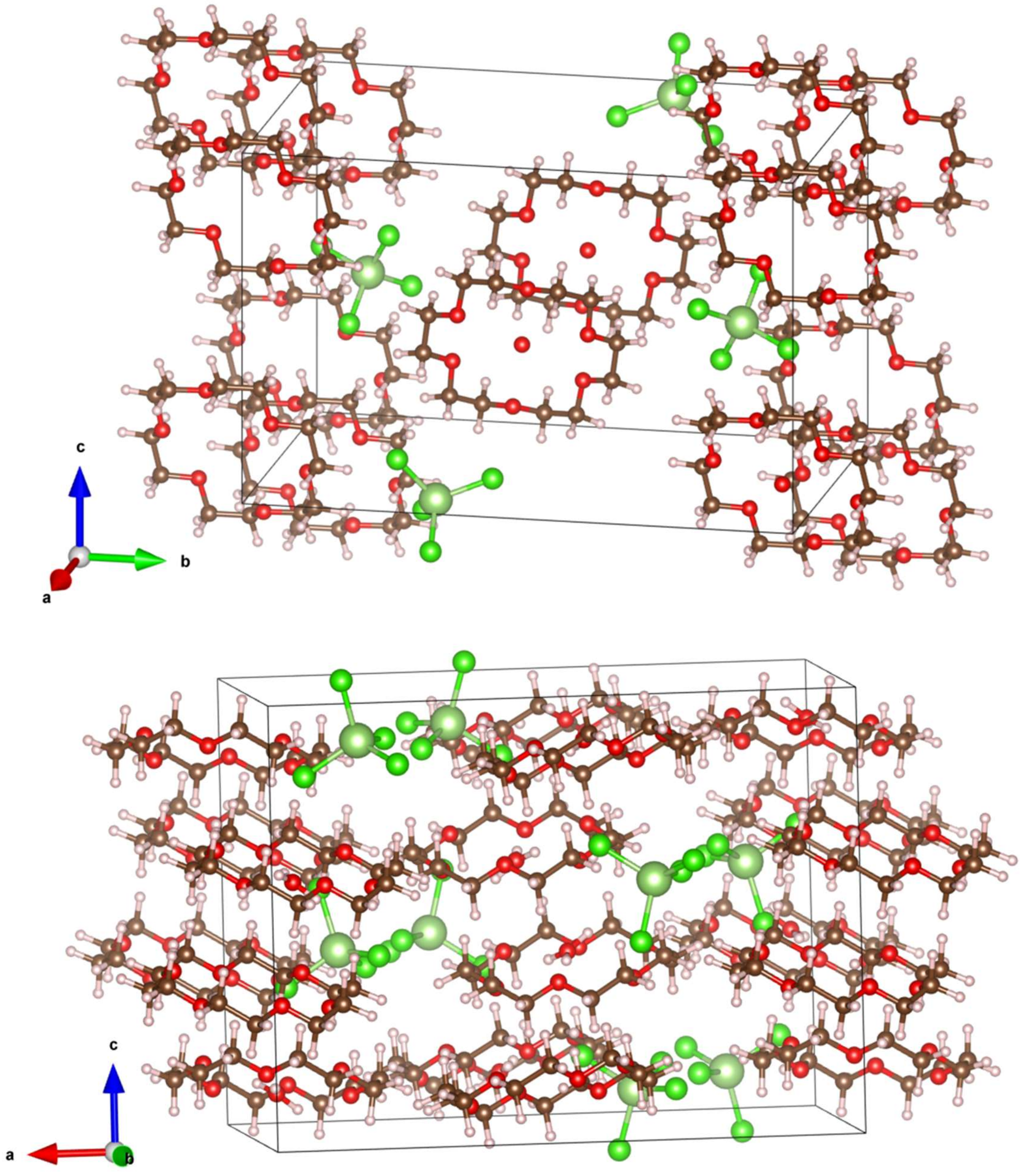

Figure S5 - Crystal structure of **1**-Y (top) and **1**-X (bottom).

Table S1 - Known structures of the $[MX_n]^{-}$·18-crown-6·H_3O^{+}. 1. NbF_6^{-} and TaF_6^{-} were reported at 123 K. 2. $AuCl_4^{-}$ was reported at 100 K.

**Counter ion**	**Ambient polymorph**	**Reported polymorphs**	**Reference**
$GaCl_4^{-}$ (**1**)	A	A, D, X, Y, W	1, this work
$FeCl_4^{-}$	B	B, Y, D	2, 3
PF_6^{-}	C	*Pnma, P*$2_1/c$	4
NbF_6^{-}, TaF_6^{-}, AsF_6^{-}	C		5, 6
BF_4^{-}	$P4_12_12$		7
$AuCl_4^{-}$	C	$P2/m$	8
$ReCl_6^{-}$, $SbCl_6^{-}$	$\bar{R}3$		9, 10
$TaCl_6^{-}$	$R3$		11

Table S2 - Crystal data and structure refinement of **1**-Y PXRD data.

**Empirical formula**	$C_{12}H_{27}O_7GaCl_4$	**Radiation**	Mo *Kα*
**Formula weight**	494.872	**2*ϑ* / °**	2-35
**Space group**	Monoclinic, $P2_1/n$	***T* / *K***	281
**Unit cell dimensions**		$\mathbf{B_{eq}}$ **/ Å^2**	
a / Å	8.6732(6)	Ga	7.1(3)
b / Å	20.7255(14)	18-crown-6	11.8(5)
c / Å	12.7011(10)	H_3O^+	3.8(11)
β / °	103.250(3)	$\mathbf{R_{wp}}$ **(%)**	5.81
***V* / Å^3**	2222.3(3)	$\mathbf{R_{Bragg}}$ **(%)**	4.62
Z	4	$\mathbf{R_p}$ **(%)**	4.97
$\mathbf{D_x}$ **/ g cm^{-3}**	1.47005 (18)	**GOF**	3.11
**Reflections**	1486		
**Parameters**	50		

Table S3 - Crystal data and structure refinement of **1**-X PXRD data.

**Empirical formula**	$C_{12}H_{27}O_7GaCl_4$	**Radiation**	Mo *Kα*
**Formula weight**	494.872	**2*ϑ* / °**	2-35
**Space group**	Orthorhombic, *Pbca*	***T* / *K***	87
**Unit cell dimensions**		$\mathbf{B_{eq}}$ **/ Å^2**	
a / Å	20.1509(17)	Ga	0.8(2)
b / Å	13.3264(12)	18-crown-6	3.9(5)
c / Å	15.2078(13)	H_3O^+	0.7(11)
***V* / Å^3**	4083.9(6)	$\mathbf{R_{wp}}$ **(%)**	11.21
Z	8	$\mathbf{R_{Bragg}}$ **(%)**	6.25
$\mathbf{D_x}$ **/ g cm^{-3}**	1.6098(2)	$\mathbf{R_p}$ **(%)**	8.33
**Reflections**	1369	**GOF**	5.41
**Parameters**	48		

Table S4 - Crystal data and structure refinement of **1**-D PXRD data.

**Empirical formula**	$C_{12}H_{27}O_7GaCl_4$	**Radiation**	Mo *Kα*
**Formula weight**	494.872	**2*ϑ* / °**	2-35
**Space group**	Tetragonal, *P4/mbm*	***T* / *K***	398
**Unit cell dimensions**		$\mathbf{B_{eq}}$ **/ Å^2**	
a / Å	10.3782(3)	Ga	26.7(9)
c / Å	11.1734(5)	18-crown-6	13(5)
***V* / Å^3**	1203.45(8)	H_3O^+	17(4)
Z	2	$\mathbf{R_{wp}}$ **(%)**	6.09
$\mathbf{D_x}$ **/ g cm^{-3}**	1.426(9)	$\mathbf{R_{Bragg}}$ **(%)**	2.07
**Reflections**	243	$\mathbf{R_p}$ **(%)**	4.47
**Parameters**	36	**GOF**	2.76

Table S5 - Crystal data and structure refinement of 18-crown-6 oxonium tetrachloro-iron(III) SXRD data.

Crystal data			
**Empirical formula**	$C_{12}H_{27}O_7FeCl_4$	***V* / Å^3**	2255.40(18)
**Formula weight**	480.994	**D_x / g cm^{-3}**	1.414
**Space group**	Monoclinic, $P2_1/n$	**Radiation**	Mo *Kα*
**Unit cell dimensions**		**2ϑ / °**	4-52
a / Å	8.7508(4)	***T* / *K***	345
b / Å	20.1464(10)	**μ / mm^{-1}**	1.168
c / Å	12.7982(6)	**Crystal size / mm^3**	0.42 × 0.252 × 0.188
β / °	103.904(2)	***Z***	4
Refinement			
**Refinement on *F*^{2}**		***R*** (I > 2σ)	0.1073
**Independent Reflections**	4435	**R_w** (I > 2σ)	0.3337
**Parameters**	218		

Table S6 – Fractional coordinates of atoms determined by SXRD for 18-crown-6 oxonium tetrachloro-iron(III) at 345 K.

Atom	x	y	z
Fe1	0.54993(18)	0.65318(7)	0.47189(10)
Cl1	0.4294(4)	0.70746(14)	0.3300(2)
Cl2	0.3848(6)	0.6402(2)	0.5758(3)
Cl3	0.6159(4)	0.55937(15)	0.4242(3)
Cl4	0.7595(6)	0.70490(17)	0.5553(4)
O1	0.6753(17)	0.5053(8)	-0.1150(12)
O2	0.7755(15)	0.4116(7)	0.0388(12)
O3	0.9728(18)	0.4449(9)	0.2367(15)
O4	0.990(3)	0.5757(18)	0.303(2)
O5	0.823(2)	0.6675(9)	0.1495(18)
O6	0.6375(18)	0.6271(8)	-0.0393(13)
C1	0.648(3)	0.4458(12)	-0.1170(18)
H1A	0.617622	0.433307	-0.1922
H1B	0.555102	0.440036	-0.08875
C2	0.759(2)	0.4010(9)	-0.0649(15)
H2A	0.721727	0.357433	-0.0835
H2B	0.857718	0.407067	-0.08491
C3	0.885(2)	0.3709(11)	0.1025(17)
H3A	0.848878	0.326715	0.090853
H3B	0.984302	0.374446	0.081847
C4	0.907(2)	0.3859(10)	0.2087(17)
H4A	0.806289	0.38419	0.22747
H4B	0.974301	0.353334	0.250884
C5	1.003(3)	0.4663(11)	0.3328(19)
H5A	1.071693	0.435696	0.379059
H5B	0.906029	0.468518	0.356132

C6	1.075(4)	0.5271(15)	0.348(2)
H6A	1.109449	0.535308	0.424256
H6B	1.169139	0.525544	0.31963
C7	1.006(3)	0.6321(16)	0.304(2)
H7A	1.093405	0.641239	0.271838
H7B	1.037354	0.645387	0.378281
C8	0.886(4)	0.6711(14)	0.255(3)
H8A	0.801684	0.664518	0.290644
H8B	0.922595	0.715039	0.269933
C9	0.702(3)	0.6996(11)	0.1091(18)
H9A	0.717156	0.74477	0.128203
H9B	0.612043	0.683659	0.133697
C10	0.678(2)	0.6912(9)	-0.0022(17)
H10A	0.773168	0.70375	-0.02295
H10B	0.594529	0.720057	-0.03826
C11	0.623(3)	0.6154(11)	-0.1347(18)
H11A	0.722738	0.622442	-0.15323
H11B	0.546326	0.64469	-0.17738
C12	0.571(3)	0.5480(10)	-0.1608(17)
H12A	0.474316	0.540512	-0.1383
H12B	0.548359	0.542135	-0.23824
O1W	0.8216(11)	0.5375(5)	0.0972(7)
H1WA	0.873427	0.548855	0.159458
H1WB	0.819757	0.497055	0.080258
H1WC	0.780697	0.562185	0.045378

References

1. H. Y. Zhang, S. Q. Lu, X. Chen, R. G. Xiong and Y. Y. Tang, *Chemical Communications*, 2019, **55**, 11571-11574.
2. H. T. Zhou, C. F. Wang, Y. Liu, X. W. Fan, K. Yang, W. J. Wei, Y. Z. Tang and Y. H. Tan, *Chemistry-an Asian Journal*, 2019, **14**, 3946-3952.
3. P. C. Junk and J. L. Atwood, *Journal of Chemical Crystallography*, 1994, **24**, 247-250
4. Y. Z. Tang, Z. F. Gu, C. S. Yang, B. Wang, Y. H. Tan and H. R. Wen, *Chemistryselect*, 2016, **1**, 6772-6776.
5. M. S. Fonari, Y. A. Simonov, W. J. Wang, S. W. Tang, E. V. Ganin, V. O. Gelmboldt, T. S. Chernaya, O. A. Alekseeva and N. G. Furmanova, *Polyhedron*, 2007, **26**, 5193-5202.
6. H. Feinberg, I. Columbus, S. Cohen, M. Rabinovitz, H. Selig and G. Shoham, *Polyhedron*, 1993, **12**, 1811-1816.
7. H. Feinberg, I. Columbus, S. Cohen, M. Rabinovitz, H. Selig and G. Shoham, *Polyhedron*, 1993, **12**, 2913-2919.
8. M. Calleja, K. Johnson, W. J. Belcher and J. W. Steed, *Inorganic Chemistry*, 2001, **40**, 4978-4985.
9. L. J. Barbour, L. R. MacGillivray and J. L. Atwood, *Journal of Chemical Crystallography*, 1996, **26**, 59-61.
10. B. Neumuller, M. Plate and K. Dehnicke, *Zeitschrift Fur Kristallographie*, 1994, **209**, 92-92.

11. B. M. Bulychev and V. K. Bel'sky, *Zhurnal Neorganicheskoi Khimii*, 1995, **40**, 1834.
